# In Vivo Detection of Circulating Tumor Cells in High-Risk Non-Metastatic Prostate Cancer Patients Undergoing Radiotherapy

**DOI:** 10.3390/cancers11070933

**Published:** 2019-07-03

**Authors:** Shukun Chen, Gerlinde Tauber, Tanja Langsenlehner, Linda Maria Schmölzer, Michaela Pötscher, Sabine Riethdorf, Andra Kuske, Gerd Leitinger, Karl Kashofer, Zbigniew T. Czyż, Bernhard Polzer, Klaus Pantel, Peter Sedlmayr, Thomas Kroneis, Amin El-Heliebi

**Affiliations:** 1 Department of Laboratory Medicine, Nanfang Hospital, Southern Medical University, Guangzhou 510515, China; 2 Department of Cell Biology, Histology and Embryology, Gottfried Schatz Research Center, Medical University of Graz, 8010 Graz, Austria; 3 Department of Therapeutic Radiology and Oncology, Medical University of Graz, 8036 Graz, Austria; 4 Institute of Tumor Biology, University Medical Center Hamburg-Eppendorf, 20246 Hamburg, Germany; 5 Diagnostic and Research Institute of Pathology, Medical University Graz, 8036 Graz, Austria; 6 Division Personalized Tumor Therapy, Fraunhofer Institute for Toxicology and Experimental Medicine, 93053 Regensburg, Germany; 7 Center for Biomarker Research, CBmed, 8010 Graz, Austria

**Keywords:** circulating tumor cells, in vivo detection, non-metastatic prostate cancer, radiotherapy

## Abstract

High-risk non-metastatic prostate cancer (PCa) has the potential to progress into lethal disease. Treatment options are manifold but, given a lack of surrogate biomarkers, it remains unclear which treatment offers the best results. Several studies have reported circulating tumor cells (CTCs) to be a prognostic biomarker in metastatic PCa. However, few reports on CTCs in high-risk non-metastatic PCa are available. Herein, we evaluated CTC detection in high-risk non-metastatic PCa patients using the in vivo CellCollector CANCER01 (DC01) and CellSearch system. CTC counts were analyzed and compared before and after radiotherapy (two sampling time points) in 51 high-risk non-metastatic PCa patients and were further compared according to isolation technique; further, CTC counts were correlated to clinical features. Use of DC01 resulted in a significantly higher percentage of CTC-positive samples compared to CellSearch (33.7% vs. 18.6%; *p* = 0.024) and yielded significantly higher CTC numbers (range: 0–15 vs. 0–5; *p* = 0.006). Matched pair analysis of samples between two sampling time points showed no difference in CTC counts determined by both techniques. CTC counts were not correlated with clinicopathological features. In vivo enrichment using DC01 has the potential to detect CTC at a higher efficiency compared to CellSearch, suggesting that CTC is a suitable biomarker in high-risk non-metastatic PCa.

## 1. Introduction

Prostate cancer (PCa) remains the most commonly diagnosed malignancy and one of the top leading causes of cancer-related death in men in developed countries [1]. In localized disease, PCa is slow-growing cancer with a 5-year survival rate of >99% [1]; yet, once the disease has progressed, the overall survival is dismal, with a 5-year survival rate of only 30% [1]. A high-risk group of PCa, defined by stage T2c or prostate-specific antigen (PSA) levels ≥ 20 ng/mL or Gleason score ≥ 8 [2], has the most potential to develop biochemical progression, local recurrence, or even metastatic disease. Treatment of high-risk non-metastatic PCa patients includes androgen deprivation therapy, radical prostatectomy, and/or radiotherapy, but there remains uncertainty as to which approach is superior due to the absence of surrogate biomarkers [3]. Thus, there is an urgent clinical need to define an early detection biomarker for the identification of high-risk non-metastatic PCa patients with the highest probability of developing disease progression. PSA level is one of the most widely used markers for the initial screening and therapy monitoring of PCa in routine clinical practice. However, PSA is not cancer-specific, as its level may also be elevated in men with benign prostatic hyperplasia [4]. Furthermore, some PCa patients were found not to have an elevated PSA [5]. Thus, given the limited specificity and sensitivity of PSA, research on the improvement of other suitable biomarkers is ongoing.

**Figure 1:**
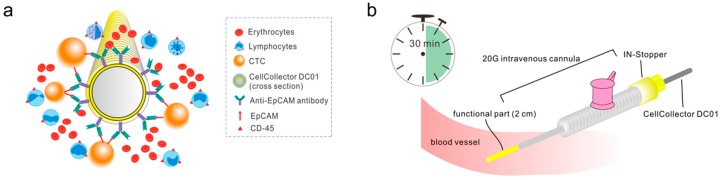
Schematic drawing of the CellCollector CANCER01 (DC01) and the application procedure. (**a**) Working principles of DC01 viewed from its cross section. A medical wire coated with hydrogel is functionalized with antibodies against epithelial cell adhesion molecule (EpCAM). Cancer cells expressing EpCAM antigen are targeted and captured onto the functional part. (**b**) Demonstration of application procedure of DC01 in vivo. The DC01 is applied into the cubital vein of the patient via a 20-gauge intravenous cannula fixed with a yellow IN-Stopper. The functional part is exposed to the blood flow for 30 min allowing the efficient detection of potential circulating tumor cells (CTCs).

Circulating tumor cells (CTCs), a group of cancer cells escaping from the primary tumor or metastases into the peripheral circulation, are thought to be precursors for neoplasm metastasis [6,7]. The predictive and prognostic roles of CTC numbers in the setting of metastatic castration-resistant prostate cancer (mCRPC) has been standardized and validated. Data from several clinical trials suggested that a pretreatment CTC count of ≥5 may be an independent predictor of poor prognosis and be related to higher PSA levels, anemia, more liver disease, higher alkaline phosphatase levels, and bone pain symptoms in mCRPC patients [8,9,10,11]. However, CTC enrichment from peripheral blood has long proven a major challenge, given that they are extremely rare, with an estimated abundance of one CTC per mL of blood [9]. Thus, an increasing number of technological platforms have been developed to capture CTCs from blood samples of cancer patients for enumeration based on their physical and/or biological features [12,13]. To date, the CellSearch system (Menarini-Silicon Biosystems, San Diego, CA, USA) is the only Food and Drug Administration (FDA)-approved device for CTC enumeration in clinical use. This system enriches CTCs by epithelial cell adhesion molecule (EpCAM) antibody-coated ferromagnetic beads and identifies CTCs of epithelial origin (CD45^−^, DAPI^+^, and cytokeratin 8/18/19^+^) [14]. To overcome the low abundance of CTCs, an in vivo enrichment approach was reported using the CellCollector CANCER01 (DC01) [15]. Unlike other methods, which are limited by the volume of the blood sample, the DC01 device allows the in vivo isolation and enumeration of CTCs directly from the bloodstream by placing the detector via a cannula into the peripheral vein of cancer patients, thus capturing EpCAM-positive CTCs (Figure 1). In a study on lung cancer, 58% and 27% of patients were detected positive for CTCs (≥1 CTC) using the DC01 and CellSearch systems, respectively, showing that DC01 is more sensitive and efficient for CTC detection [16]. In prostate cancer, Kuske et al. [17] reported that the DC01 device enabled CTC detection in high-risk non-metastatic PCa patients treated with surgery, but data on patients treated with radiotherapy is currently lacking.

The aim of the present study was to evaluate whether CTCs can be detected in high-risk non-metastatic PCa patients before and after radiotherapy and to evaluate baseline CTC status with respect to pretreatment clinical-pathological features, including clinical T stage, PSA value, and Gleason score. We present a single center study designed to analyze CTCs using the DC01 and CellSearch system. We further compared the performance of both CTC detection platforms in CTC detection.

## 2. Results

### 2.1. Patient and Sample Characteristics

**Figure 2:**
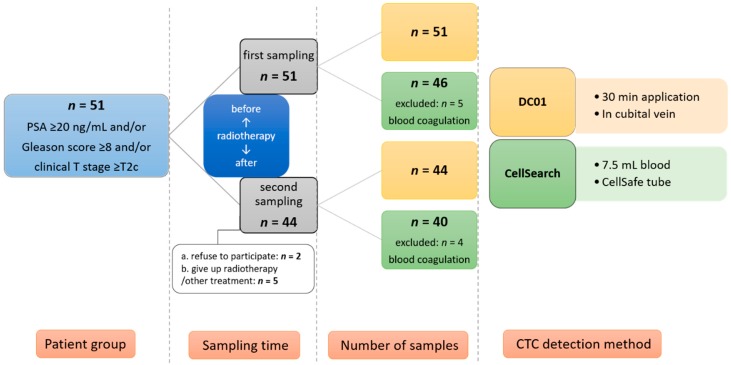
Illustration of study design and patient recruitment. CTC: circulating tumor cell.

The study was conducted from November 2013 to April 2017 and involved 51 patients with high-risk non-metastatic PCa aged 55–80 years (median, 74 years) at entry. The patients’ median PSA level was 12.0 ng/mL, and their median Gleason score was 8. A summary of the clinical features of all 51 patients at baseline is shown in Table 1. CTC status was evaluated 1 day before commencing neoadjuvant hormonal therapy (first sampling); 44 patients underwent a second sampling 3 months after completion of radiotherapy (Figure 2). Of the patients who didn’t visit the radiotherapy department for the second sampling, two left the study after radiotherapy, four canceled radiotherapy (one due to the development of bone metastasis), and one patient developed second cancer and received a different treatment. Till now, none of the patients included in the present investigation has developed biochemical or clinical relapse at the follow-up visit. 

Among the 44 patients who underwent the second sampling, all of them had paired samples for DC01 analysis. CellSearch analysis of four blood samples at the second sampling time point was failed due to the blood coagulation. Among the remaining 40 patients, four patients didn’t have CTC status detected by CellSearch at the first sampling time point due to the blood coagulation. Thus, 36 of them had paired blood samples for CellSearch analysis.

### 2.2. Detection of CTCs and CTC Clusters Using DC01

**Figure 3:**
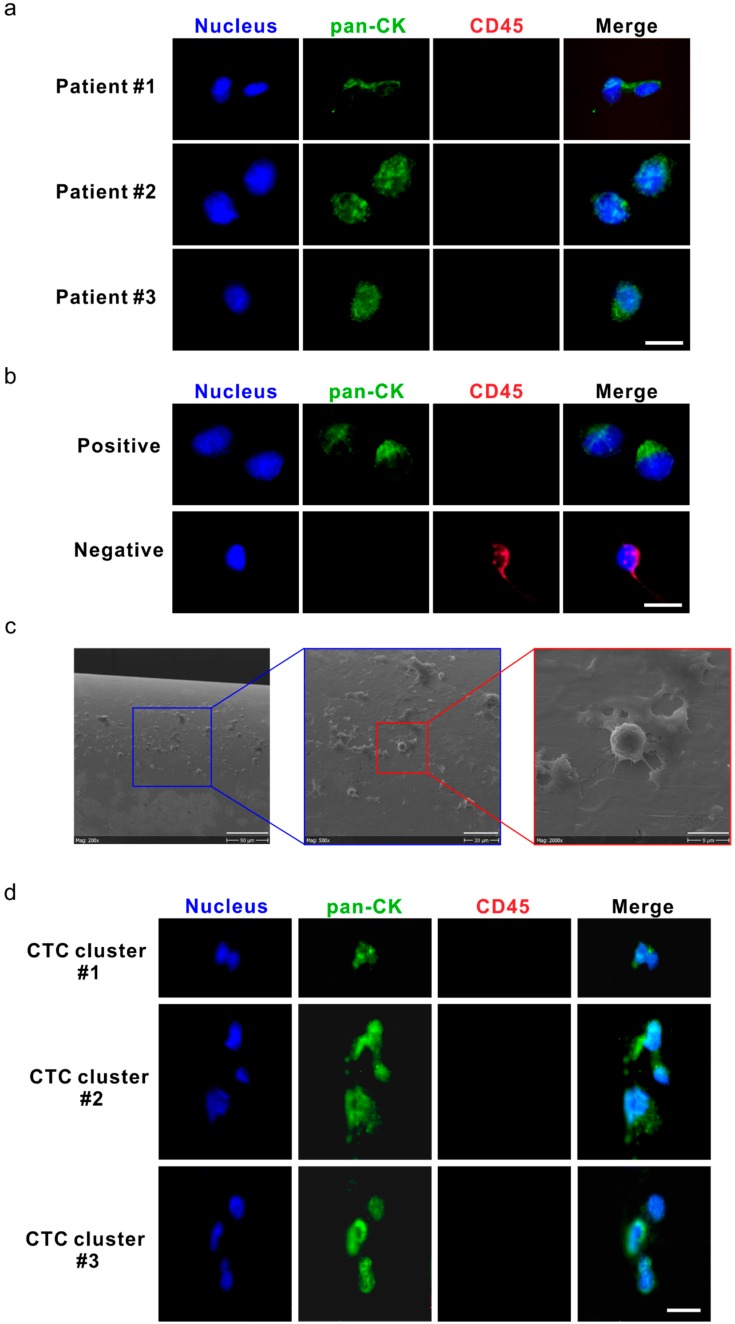
Circulating tumor cells (CTCs) and CTC clusters were detected by the DC01 device in patients with high-risk non-metastatic prostate cancer (PCa). (**a**) Micrographs of five CTCs on the DC01 detected from three patients; CTCs were defined as being positive for nucleic staining and pan-CK (pan-cytokeratin), and negative for CD45 by immunofluorescence staining (DNA (blue) and pan-CK (green)). Scale bar: 20 μm. (**b**) Images of control samples for CTC staining panel, upper panel: LNCaP cells captured on the DC01 as positive control for CTC staining panel by immunofluorescence staining (DNA (blue) and pan-CK (green)); lower panel: leukocytes attached to the DC01 served as negative controls for the CTC staining panel by immunofluorescence staining (DNA (blue) and CD45 (red)). Scale bar: 20 μm. (**c**) Scanning electron microscope image showing the surface of DC01 and blood components, including unspecific binding of leucocytes onto DC01. Scale bar (from left to right): 90 μm, 30 μm, and 9 μm. (**d**) Three CTC clusters on the DC01 detected from two patients; CTC clusters were found as a cluster of CTCs being positive for nucleic staining and pan-CK, and negative for CD45 by immunofluorescence staining (DNA (blue) and pan-CK (green)). All CTCs were negative for PSA, so this channel is not shown. Exposure time for each channel: nucleus DNA, 800~1000 ms; pan-CK, 3000 ms; CD45, 6000 ms. Scale bar: 20 μm.

The short-term application of DC01 in vivo for 30 min was well tolerated in all patients without any side effects. Representative images of collector-captured CTCs are depicted in Figure 3a. All verified CTCs were positive for the expression of pan-cytokeratin (pan-CK; green) and negative for CD45 (red), with an intact nucleus (Figure 3a); LNCaP (human prostate cancer cell line) cells were used as a positive control for the staining (Figure 3b, upper panel). Additionally, lymphocytes not specifically bound on the collector were used as an internal negative control (Figure 3b, lower panel, Figure 3c). The size of CTCs varied from 4 μm to 20 μm. We identified three CTC clusters, each containing 2–3 CTCs, in samples from two patients, with total CTC counts of 10 and 15, respectively (Figure 3d). 

### 2.3. Analysis of CTC Status at Baseline

Evaluation of the DC01 revealed that 39.2% (20/51) of patients were positive for CTCs (Table 2). Clinical features (i.e., T stage, Gleason score, and baseline PSA level) were stratified by CTC status (Table 1). The Mann–Whitney *U* test showed that there was no significant difference in clinicopathological factors (T stage, PSA value, Gleason score) between CTC-positive and CTC-negative patient groups (Table 1). Clinical parameters and CTC counts of the CTC-positive patients are compiled in Table 3. Importantly, application of DC01 in two healthy individuals did not show the capturing of EpCAM-positive cells, as also reported by others [16]. 

### 2.4. Analysis of Paired Blood Samples Before and After Radiotherapy Using the Two Methods

**Figure 4:**
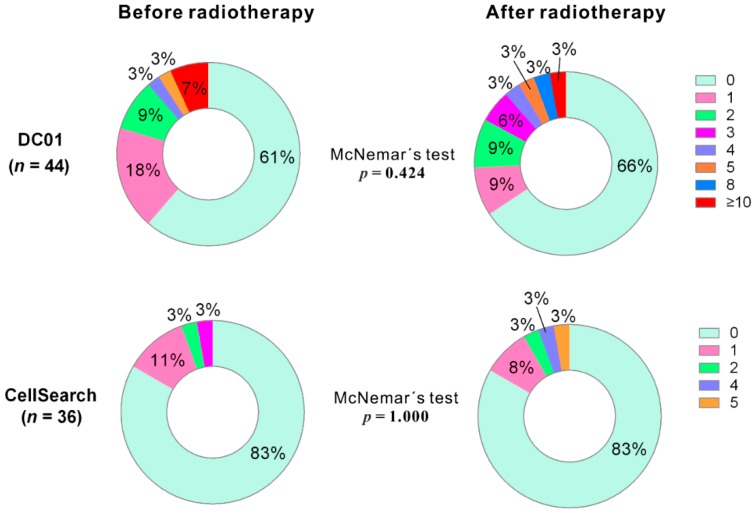
Distribution of circulating tumor cell (CTC) counts detected by DC01 and CellSearch at two sampling time points (i.e., 1 day before commencing radiotherapy and 3 months after completion of radiotherapy) (*p* < 0.05 statistically significant, two-tailed, McNemar’s test).

We analyzed CTC counts of paired samples before and after radiotherapy using both detection methods (for DC01, *n* = 44; for CellSearch, *n* = 36). For DC01, although the percentage of CTC-positive samples decreased from 39.2% to 27.3% (Table 2), McNemar’s test indicated that no statistically significant reduction in the percentage of CTC-positive samples detected after treatment (*p* = 0.424) (Figure 4, upper panel). Similarly, no reduction in CTC-positive samples was observed after treatment in samples processed with CellSearch (*p* = 1.000) (Figure 4, lower panel). 

### 2.5. DC01 Improves CTC Enrichment Efficiency in A Cohort of High-Risk Non-Metastatic PCa Patients

We assessed the baseline CTC status of patients by comparing CTC detection using DC01 (*n* = 51) and CellSearch (*n* = 46). DC01 reported almost twice as many patients to be CTC positive (39.2%, 20/51) compared to CellSearch (19.6%, 9/46). In addition, the number of CTCs per patient detected by DC01 (range 0–15; median = 0; 75th percentile = 1) was higher compared to that detected by CellSearch (range 0–3; median = 0; 75th percentile = 0) (Table 2). From the 44 samples collected on the second measurement after radiotherapy, four samples could not be analyzed by CellSearch due to blood coagulation (*n* = 4). On the second measurement, 27.3% (12/44) and 17.5% (7/40) of patients were CTC positive using DC01 and CellSearch, respectively; similarly, the number of CTCs detected by DC01 (range 0–14; median = 0; 75th percentile = 1) were higher compared to those detected by CellSearch (range 0–5; median = 0; 75th percentile = 0).

**Figure 5:**
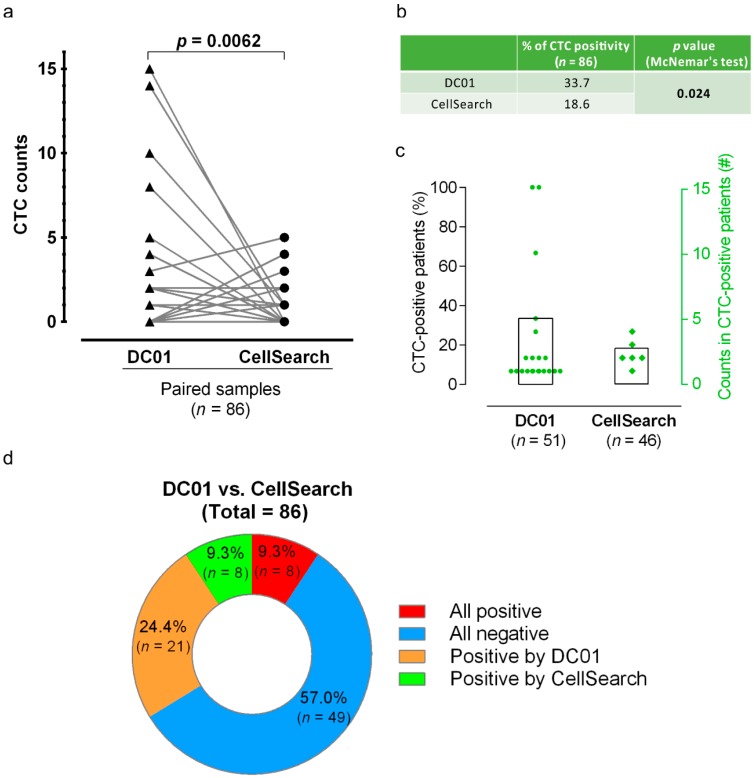
Comparison of circulating tumor cell (CTC) positivity and CTC counts examined by the two CTC detection methods. (**a**) Comparison of CTC numbers by parallel analysis of patient samples using DC01 and CellSearch. The plot links the number of CTCs detected by means of parallel enumeration using DC01 and CellSearch. DC01 detected significantly higher numbers of CTCs than CellSearch (Wilcoxon matched-pairs signed-rank test, two-tailed, *p* = 0.0062). (**b**) Comparison of CTC positivity of both methods (*p* < 0.05 statistically significant, McNemar’s test). (**c**) Percentage of CTC-positive patients (bars, 33.7% and 18.6%) and number of CTCs detected per patient (dots, squares) listed for the parallel analysis in 51 patients using DC01 and CellSearch, respectively. (**d**) Concordance between the two CTC detection methods. Assays were not concordant if CTC status of one sample was differently detected by each method, meaning one assay was positive and the other negative (orange/green). Assays were assumed as concordant when CTC status was the same for one sample detected by both techniques (all positive <red> or all negative <blue>).

Paired analysis across all 86 samples by combining the results of CTC count from the two sampling points showed that DC01 detected higher CTC counts than CellSearch (Wilcoxon matched-pairs signed-rank test, two-tailed, *p* = 0.0062) (Table 4, Figure 5a). Additionally, the percentage of CTC-positive patients identified by DC01 was higher compared to that identified by CellSearch (33.7% vs. 18.6%, McNemar’s test, *p* = 0.024) (Figure 5b,c).

Finally, concordant between the two methods in detection of CTC-positive samples were obtained in eight samples (9.3%), while 49 samples lacked CTCs through both analysis methods (57.0%) (Figure 5d).

### 2.6. Conversions of CTC Status after Radiotherapy

A total of 44 patient samples were analyzed with DC01 before and after therapy. The CTC status of 19 patients did not change, with 17 patients remaining CTC negative and two patients remaining CTC positive. Of the remaining 25 patients for whom CTC status changed, 10 switched from negative to positive (range 1–14 CTCs) and 15 from positive to negative status (Table 5). 

Among the 36 paired samples available for CellSearch analysis, no change in CTC status was observed for 24 patients, all of whom remained CTC negative. Further, all six CTC-positive patients changed to CTC-negative status after therapy, whereas six CTC-negative patients changed to positive CTC status (range 1–5 CTCs) after therapy (Table 5).

## 3. Discussion

We evaluated the feasibility of detecting CTC numbers and monitoring these in high-risk non-metastatic PCa patients undergoing radiotherapy. Our data show that CTCs can be detected using both the in vivo DC01 and the CellSearch system before and after radiotherapy. This feasibility study demonstrates that CTCs in PCa can be detected even without metastatic disease and, therefore, that CTC monitoring has the potential to support early detection of disease progression in high-risk non-metastatic PCa.

Despite major advances in the treatment of metastatic cancer, the most effective way to reduce cancer mortality is early detection prior to progression when cancer remains localized within the primary site. There is considerable evidence that early detection of cancer relapse or progression is associated with greater treatment options and better prognosis [18,19,20]. Implementation of liquid biopsies for the early detection of localized cancer is highly challenging as CTCs and circulating tumor DNA are less abundant than in metastatic disease and require highly sensitive methods [21,22,23]. A promising approach to increase the likelihood of CTC detection is tumor-derived extracellular vesicles, which show a 20 times higher frequency in blood compared to CTCs and provide similar prognostic information to CTC counts [24]. Similarly, Lambros et al. [25] developed an apheresis protocol to increase the CTC yield by up to 100-fold, which may permit the isolation of CTCs in a localized disease stage where a low number of CTCs are present in the blood. In our study, we employed the in vivo DC01 device for CTC detection, which substantially increases the chance for CTC isolation compared to other technologies based on ex vivo analysis in a limited volume of sampled blood. During the 30 min of in vivo application, it is estimated that 1.5 to 3 L of blood pass by DC01, enabling the capture of CTCs [15]. The presence of CTCs detected by DC01 in high-risk non-metastatic PCa was consistent with previous studies in early-stage cancer disease [26,27,28,29] and supports findings that metastatic dissemination is an early event in tumor progression [30]. 

Compared to single CTCs, CTC clusters may be more aggressive in forming distal metastasis [31,32]. Previous studies have demonstrated that patients with detectable CTC clusters have significantly worse prognosis or develop resistance to therapy [31]. Several specialized platforms have been developed to isolate CTC clusters [33,34,35]. Despite DC01 not having been designed to capture CTC clusters, we observed three CTC clusters, each comprised of two to three CTCs, in two patients who also presented with higher CTC numbers. Similarly, using DC01, Gorges et al. [36] identified CTC clusters (range 3–7 cells) in 20 out of 185 lung cancer patients. The low incidence of CTC clusters in our study might be ascribed to the fact that all patients had non-metastatic localized cancer.

The detection of CTCs as a prognostic marker has been widely studied in metastatic PCa [8,9,24,37]. However, in localized PCa, only a few studies have been performed, with practically all using CellSearch. Using the cut-off value of one cell, a CTC positivity rate of within 5% and 27% was observed [17]. As CellSearch and DC01 identify CTCs based on EpCAM and cytokeratin expression, we performed a direct comparison of both methods. Our data showed that the incidence of CTC positivity at baseline using CellSearch was 19.6%, which was within the range of published data by other studies assessing non-metastatic PCa [17,38,39,40,41,42]. Our data show higher pretherapy CTC detection rates of up to 39.2% (20/51 patients, range 1–15 CTCs) compared to published data, with the gold standard CTC isolation method CellSearch showing a rate of 19.6% positivity (9/46 patients, range 1–3 CTCs). We further combined the results of CTC counts from both sampling time points and revealed that DC01 showed a significantly increased detection rate compared with CellSearch (33.7% vs. 18.6%; *p* = 0.024). Besides a higher percentage of CTC-positive samples, paired analysis displayed a significantly higher number of CTC counts using DC01 compared to CellSearch (Wilcoxon matched-pairs signed-rank test, two-tailed, *p* = 0.0062), showing that DC01 may be a more effective tool for CTC isolation. A similar comparison was performed by Gorges et al. in 2015 [36], who demonstrated a higher CTC detection efficiency using DC01 in lung cancer patients. For the purpose of using CTC as a potential biomarker in localized PCa, larger patient cohorts and long-term follow-up information are critical with respect to PSA response, PSA progression-free survival, progression-free survival, and overall survival. Furthermore, comparative studies are also necessary to assess each potential valuable platform for capturing CTC with high sensitivity and specificity. Nevertheless, the prognostic relevance of the DC01 device needs to be further evaluated with long- term patient data.

We observed no statistical correlations between the presence of CTCs at baseline and a series of clinicopathological features typically used to predict cancer disease outcome. This is in line with the currently limited literature that demonstrates low CTC numbers detected using the CellSearch system in high-risk non-metastatic PCa [16,41,42,43,44], suggesting that CTC provides prognostic information independent of currently used parameters for evaluating cancer status. We did not find any clinical features indicating the worse status of cancer progression in CTC-positive patients. Furthermore, we found no statistical difference with respect to clinical characteristics between CTC-positive and CTC-negative patient groups. Notably, several patients showed very high-risk of cancer status based on conventional pathological features; they all had either a T stage of three, and/or PSA value more than 60 ng/mL, and/or Gleason score of ≥9. However, they were all found to be negative for CTC using both detection methods. Several studies have reported that CTC-negative patients have better clinical outcomes. Thus, further follow-up of clinical outcomes is crucial to explore whether these patients can be more successfully treated with radiotherapy compared to CTC-positive patients.

Due to the increased potential of high-risk non-metastatic PCa to develop recurrence and metastasis, it is crucial to take effective action to confine disease. At present, the most important treatment for high-risk non-metastatic PCa is radical prostatectomy and radiotherapy, but no consensus has yet been reached on the best course of treatment. Recently, Parker et al. [45] reported a randomized trial on the effect of radiotherapy to the metastatic PCa. They concluded that radiotherapy should be a standard treatment option for men with a low metastatic burden [45], although some researchers suggested that the definition of a low metastatic burden in their study is not standard and should be further modified [46]. Additionally, it is still unknown whether radical prostatectomy can be a comparable treatment option [47]. For PCa patients in whom micrometastases are failed to be detected by current imaging tools, radiotherapy may not provide enough benefit to each of them due to the existence of the minimal residual disease, and a proportion of patients will develop biochemical recurrence by an increasing PSA level [48]. Therefore, it is crucial to identify those patients who would benefit from more aggressive treatment using liquid biopsies able to predict response to radiotherapy. A pilot study by Lowes et al. [49] demonstrated that CTC enumeration by CellSearch might be valuable in the stratification of patients regarding the level of treatment benefit before the initiation of radiotherapy. Further follow-up studies are necessary to evaluate the potential of CTC as a surrogate prognostic marker for cancer progression as well as a predictive marker of response to radiotherapy. 

PCa has been shown to be a highly heterogeneous cancer with complex clinical and molecular behaviors [50,51,52], which largely determine the status and treatment options [53,54,55,56]. Thus, beyond CTC enumeration, CTC molecular characterization provides an additional approach to monitor disease progression and optimize therapeutic care in individual cancer patients for personalized medicine. Several genetic alterations have been reported as driver event of cancer progression, drug resistance, and relapse, including androgen receptor (AR) variants [57,58], Phosphatase and tensin homolog (PTEN) loss [59,60], and E26 transformation–specific (ETS) gene rearrangement [61,62], among others, as assessed mainly based on tissue samples from tumor entities. Undoubtedly, using conventional tumor biopsies to detect and characterize cancer remains the golden standard. However, for monitoring purposes, tissue biopsies by fine-needle aspiration remain unfeasible due to the invasiveness of the procedure. Therefore, liquid biopsy offers an alternative resource wherein samples can be rapidly acquired non-invasively with less pain, risk, and expense [63].

Additionally, since CTCs carry genetic information from either the primary tumor or the metastasis site, further molecular analysis can be performed [64,65,66]. Recent studies on mCRPC demonstrated that molecular analysis of CTCs could serve as predictive biomarkers for clinical response to hormonal therapies. One of such targets is the androgen receptor splice variant- 7 (AR-V7) mRNA of CTCs in mCRPC. It has been shown that detectable AR-V7 mRNA or protein in CTCs are associated with resistance to enzalutamide- and abiraterone-based anti-androgen therapy [67,68,69]. Antonarakis et al. further characterized the prognostic significance of CTC-based AR-V7 mRNA detection by expanding the analysis in a cohort of 202 mCRPC patients receiving first-line and second-line novel hormonal therapies [70]. Their study confirmed that patients categorized as CTC^+^/AR^−^V7^+^ had worse clinical outcomes compared to those categorized as CTC^−^ and CTC^+^/AR^−^V7^−^ [70]. Recently, our group developed a padlock probe approach with the ability to visualize specific mRNA transcripts, such as AR-V7, in CTCs [71]. Using the padlock probe approach, we visualized heterogeneity among CTCs, which may be able to provide a suitable approach to quantify AR-V7 expression and CTC numbers. The importance of molecular characterization led to the development of a new type of CellCollector CANCER03 (Catch & Release, C&R) [72,73], which showed promising results in CTC detection and single CTC analysis. However, the C&R has not yet been CE certified and is currently only available for in vitro studies.

Our study aimed to evaluate the feasibility of CTC detection in high-risk non-metastatic PCa patients undergoing radiotherapy. However, a major limitation to our study is the long time required to investigate the prognostic relevance of our findings as the cancer-specific outcomes need to be evaluated for 3–5 years in high-risk non-metastatic PCa to draw feasible conclusions [74]. Nevertheless, we showed herein that the CellCollector DC01 constitutes an efficient CTC detection technology, which might enable a noninvasive tool for monitoring cancer relapse in localized disease as well as evaluation of treatment response.

## 4. Materials and Methods

### 4.1. Patient Recruitment

This study was approved by the ethics committee of the Medical University of Graz (25-240 ex 12/13). Written informed consent was obtained from all patients. We enrolled 51 high-risk non-metastatic PCa patients according to the D’Amico risk classification [2]. High-risk status was defined as PSA ≥ 20 ng/mL and/or Gleason score ≥ 8 and/or clinical T stage ≥ T2c. The patient staging was performed according to the seventh edition of the Tumor, Node, Metastasis (TNM) Classification of Malignant Tumors [75]. Before the start of treatment, all patients underwent abdominal/pelvic MRI and a bone scan to exclude metastatic disease. Patients first received neoadjuvant hormone therapy for 6–9 months followed by radiotherapy (doses of 78 Gy each time) with volumetric modulated arc therapy five times a week for 7–9 weeks plus long-term concomitant/adjuvant androgen deprivation for 2–3 years. PSA relapse was defined as a rise by ≥2ng/mL above the nadir PSA according to international guidelines [76]. In case of PSA relapse, patients are regularly evaluated with a battery of diagnostic tests, including isotope bone scan, chest X-ray, and abdominal and pelvic computed tomography, as well as pelvic magnetic resonance imaging studies, in order to detect local and/or distant recurrences. Peripheral blood samples for CTC isolation were taken at two time points (before starting radiotherapy and 3 months after completion of radiotherapy). Enumeration of CTCs was performed using DC01 and CellSearch. Two healthy individuals were subjected to CTC analysis using the DC01. The study design and information on sample collection and processing are summarized in Figure 2.

### 4.2. Sample Collection for CTC Enumeration Using Two Methods

CTCs were enumerated before and after radiotherapy (first and second sampling) using DC01 and CellSearch. For CTC detection by means of DC01, medical wires were placed into patient´s cubital vein via an intravenous catheter (20-gauge, BD Venflon™, Helsingborg, Sweden) as previously described [71]. After 30 min of incubation at room temperature, the functional part of DC01 was removed from the patients, immediately washed three times in 1× PBS (pH 7), fixed in 100% acetone for 10 min, air-dried for 5 min, and stored at −20°C until further use for CTC enumeration.

For CellSearch analysis, 7.5 mL of peripheral blood were collected in CellSave (Veridex LLC, Raritan, NJ, USA) preservative tubes and batch-shipped at room temperature to our cooperation partner (Department of Tumor Biology, University Medical Center Hamburg-Eppendorf, Hamburg, Germany) for CTC enumeration within 96 h, as previously described [17]. 

Patient samples were assigned a CTC-positive status when at least one CTC was detected [17,36,38,41,42].

### 4.3. Sample Processing and Enumeration of CTCs Using DC01 

Cells attached to the DC01 were permeabilized in 1× PBS/0.1% Triton X-100 (Sigma, St. Louis, MI, USA) for 10 min, washed three times in 1× PBS, and blocked with 1× PBS/3% BSA (w/v, Sigma) for 30 min. Immunolabeling was performed in 300 µL of PBS/3% BSA for 1 h containing the following antibodies: anti-CD45 (MEM-28, AlexaFluor647-conjugated, Exbio, dilution 1:25), anti-pan-CK (AE1/AE3 (eBiosience, San Diego, CA, USA) and C11 (Exbio, Praha, Czech Republic); AlexaFluor488-conjugated, dilution 1:50 (both)), and anti-PSA antibody (H117, AlexaFluor555-conjugated, dilution 1:80, University of Turku, Finland). Cells were counterstained with Hoechst 33342 (Sigma, 1:10,000 in 1× PBS), rinsed, and air-dried for 5 min each. Images of stained cells were acquired using a fluorescent microscope (Observer Z1, Carl Zeiss, Jena, Germany) and identified cells positive for nucleic staining/pan-CK and negative for CD45 as CTCs. Additionally, all images of candidate CTCs were re-evaluated independently by the panel of experts involved in the multi-center clinical study CTC-SCAN. All patients showing one or more CTCs were assigned to the CTC-positive patient group.

### 4.4. Statistical Methods

The association of CTC positivity with clinicopathological variables was evaluated using the χ^2^ (likelihood) test. To compare CTC counts at both sampling time points, McNemar’s test was used. To compare the efficacy of the two methods (DC01 and CellSearch) for CTC detection, the χ^2^ (likelihood) test was used. Analyses were performed by IBM SPSS Statistics version 21.0 (IBM Corp., Armonk, NY, USA). The Wilcoxon matched-pairs signed-rank test (CTC) and Mann–Whitney *U* test were performed using GraphPad Prism version 6.0 (GraphPad Software, La Jolla, CA, USA). All tests were two-sided, and a *p*-value of less than 0.05 was considered statistically significant.

## 5. Conclusions

We reported an in vivo isolation approach using an anti-EpCAM coated device (DC01), thus circumventing limitations due to batch sampling. This method proved more efficient in CTC detection from high-risk non-metastatic PCa patients compared to CellSearch. The DC01 thus has potential clinical applications, such as in non-invasive screening for monitoring of cancer relapse in localized disease and evaluation of treatment response. Nevertheless, the use of CTC, as a surrogate biomarker, to indicate cancer recurrence and response to treatment needs to be further evaluated.

## Figures and Tables

**Table 1:** Summary of clinical characteristics of high-risk PCa patients stratified by circulating tumor cell (CTC) status at first sampling time points based on the two CTC detection methods.

Parameter	Category	DC01				CellSearch			
		Total (*n* = 51)	CTC Status		*p*-Value	Total (*n* = 46)	CTC Status		*p*-Value
			Positive (*n* = 20)	Negative (*n* = 31)			Positive (*n* = 9)	Negative (*n* = 37)	
T stage	T1	24	10	14	0.121	21	7	14	0.097
	T2	15	3	12		14	1	13	
	T3	12	7	5		11	1	10	
PSA value (ng/mL)	<10	19	5	14	0.896	18	4	14	0.424
	10–20	16	8	8		15	3	12	
	>20	16	7	9		13	2	11	
Gleason score	6	1	0	1	0.579	1	0	1	0.508
	7	2	1	1		2	0	2	
	8–10	48	19	29		43	9	34	

PSA: prostate specific antigen.

**Table 2:** CTC (circulating tumor cell) status detected by the two methods before and after radiotherapy.

Time Point	CTC Positivity (≥1 CTC)	DC01	CellSearch
**first visit** **(*n* = 51)**	% of positive samples (positive #/total #)	39.2 (20/51)	19.6 (9/46)
	range	0–15	0–3
	75th percentiles	1	0
	median	0	0
**second visit** **(*n* = 44)**	% of positive samples (positive #/total #)	27.3 (12/44)	17.5 (7/40)
	range	0–14	0–5
	75th percentiles	1	0
	median	0	0

#: number of CTC.

**Table 3:** Clinical features of circulating tumor cell (CTC)-positive patients grouped by CTC count detected by DC01 at first sampling time point before radiotherapy.

CTCs Counts	Total # of Patients with CTC	Clinical Features in Detail		
		Clinical T Stage	PSA Value (ng/mL)	Gleason Score
1	12	1	10.70	8 (4+4)
		1c	51.44	8 (4+4)
		1c	21.90	7 (3+4)
		1c	14.01	10 (5+5)
		2	11.21	9 (4+5)
		2	27.70	8 (4+4)
		3a	12.90	8 (4+4)
		3a	2.90	8 (4+4)
		3a	18.73	8 (4+4)
		3a	10.50	8 (4+4)
		3b	9.00	8 (4+4)
		3b	65.00	8 (4+4)
2	4	1c	6.22	8 (4+4)
		1c	5.62	10 (5+5)
		1c	34.60	8 (4+4)
		1c	17.70	8 (4+4)
4	1	2	12.00	8 (4+4)
5	1	2b	4.92	8 (4+4)
10	1	1c	52.10	8 (4+4)
15	2	1c	10.12	9 (4+5)
		3a	30.20	8 (4+4)

#: number, PSA: prostate specific antigen.

**Table 4:** CTC (circulating tumor cell) counts of all samples (from both sampling time points) explored by the two methods.

Detection Method			DC01		Total
			Negative	Positive	
**CellSearch**	negative	Count	49	21	70
		% within CellSearch	70.0	30.0	100.0
		% within CellCollector	86.0	72.4	81.4
	positive	Count	8	8	16
		% within CellSearch	50.0	50.0	100.0
		% within CellCollector	14.0	27.6	18.6
**Total**		Count	57	29	86
		% within CellSearch	66.3	33.7	100.0
		% within CellCollector	100.0	100.0	100.0

**Table 5:** Summary of circulating tumor cell (CTC) status detected by both methods before and after radiotherapy in paired blood samples.

Detection Method	# of Patients of Different CTC Status during the Sampling Period			
	Consistently neg.	neg.→pos.	Consistently pos.	pos.→neg.
DC01 (*n* = 44)	17 (39%)	10 (23%)	2 (4%)	15 (34%)
CellSearch (*n* = 36)	24 (67%)	6 (17%)	0	6 (17%)

#: number of CTC, neg.: negative, pos.: positive.
